# 7.N. Workshop: Mental health promotion is key to making a change for children and adolescents

**DOI:** 10.1093/eurpub/ckac129.450

**Published:** 2022-10-25

**Authors:** Organised by: EUPHA-CAPH, Chair persons: Julia Dratva (EUPHA-CAPH), Jutta Lindert (EUPHA-PMH)

## Abstract

The Covid-19 pandemic highlighted the already high burden in children and adolescents and has caused an increasing mental health burden in this age group. While provision of treatment is highly relevant, this alone is unlikely to make a significant difference in reducing the burden of poor mental health and mental illness. It is elemental to promote and strengthen mental health, also knowing this will not be the last crisis to tackle. Alongside of low threshold support and prevention, health promotion can strengthen individuals’ resilience or coping mechanisms. Next to individual and target group interventions, health promotion interventions also follow a setting approach, according to which people are understood as part of the social systems in which they live and spend their time. This workshop is about mental health promotion in youth. We wish to point to the importance of health promotion in the overall aim to improve mental health. The first presentation will provide an overview of mental health promotion concepts and mechanisms, providing evidence-based arguments and examples. The second presentation focusses on the ABC-framework to develop and implement MHP initiatives developed by the WHO. The last two presentations address the setting-approach, school and family and their potential in mental health promotion. In the concluding discussion with workshop participants, we will address questions, experiences, and best practices in mental health. The aim of this workshop is three-fold:

1. Provide the back-drop and a theoretical & practical basis on mental health promotion.

2. Present both individual and setting-specific examples of mental health promotion.

3. Provide room for discussion on experience and best practice in mental health promotion in youth.

**Key messages:**

• Mental health promotion is an important element in reducing mental health burden in children and adolescents.

• Health promotion can effectively be implemented on an individual and structural level.

